# Efficacy and Safety of First-Line Nivolumab Plus Ipilimumab in Patients with Postoperative Recurrent and Inoperable Non-Small Cell Lung Cancer: A Real-World Retrospective Observational Study

**DOI:** 10.3390/medicina61060994

**Published:** 2025-05-27

**Authors:** Yuhei Kurata, Atsuto Mouri, Hisao Imai, Satoshi Endo, Kasumi Tsukamoto, Kenji Masaki, Kosuke Hashimoto, Yu Miura, Ayako Shiono, Ou Yamaguchi, Junichi Nakagawa, Kyoichi Kaira, Kunihiko Kobayashi, Hiroshi Kagamu

**Affiliations:** 1 Department of Respiratory Medicine, Comprehensive Cancer Center, International Medical Center, Saitama Medical University, Hidaka 350-1298, Saitama, Japan; iehuy904@gmail.com (Y.K.); mouria@saitama-med.ac.jp (A.M.); kmasaki@saitama-med.ac.jp (K.M.); hkosuke@saitama-med.ac.jp (K.H.); you_mi@saitama-med.ac.jp (Y.M.); ashiono@saitama-med.ac.jp (A.S.); ouyamagu@saitama-med.ac.jp (O.Y.); kkaira1970@yahoo.co.jp (K.K.); kobakuni@saitama-med.ac.jp (K.K.); kagamu19@saitama-med.ac.jp (H.K.); 2 Division of Infectious Diseases and Respiratory Medicine, Department of Internal Medicine, National Defense Medical College, Tokorozawa 359-8513, Saitama, Japan; 3 Division of Respiratory Medicine, National Hospital Organization Disaster Medical Center, Tachikawa 190-0014, Tokyo, Japan; k.shio.1519@gmail.com; 4 Division of Respiratory Medicine, Gunma Prefectural Cancer Center, Ota 373-8550, Gunma, Japan; endo-sa@gunma-cc.jp; 5 Division of Respiratory Medicine, National Hospital Organization Takasaki General Medical Center, Takasaki 370-0829, Gunma, Japan; nakagawa.jiyunichi.za@mail.hosp.go.jp

**Keywords:** advanced non-small cell lung cancer, immune checkpoint inhibitor, ipilimumab, nivolumab, postoperative recurrence

## Abstract

*Background and Objectives:* The comparative efficacy and safety of nivolumab plus ipilimumab (Nivo-Ipi) combination therapy between patients with either postoperative recurrent non-small cell lung cancer (NSCLC) or inoperable stage III/IV NSCLC have yet to be conclusively determined. *Materials and Methods:* This retrospective study reviewed the medical records of consecutive patients diagnosed with either postoperative recurrent NSCLC or inoperable stage III/IV NSCLC. Both groups, referred to as the postoperative and inoperable cohorts respectively, underwent Nivo-Ipi therapy at four Japanese medical institutions between December 2020 and November 2022. The study’s primary aim was to evaluate and compare the efficacy and safety outcomes across these two groups. *Results:* A total of 161 patients received Nivo-Ipi therapy (postoperative group, *n* = 30; inoperable group, *n* = 131). The objective response rate was comparable between the postoperative and inoperable groups (36.7% vs. 32.1%, *p* = 0.67). Median progression-free survival did not differ significantly between groups (8.9 months vs. 6.5 months, *p* = 0.14). However, median overall survival was significantly longer in the postoperative group (not reached vs. 13.0 months, *p* = 0.012). The incidence of grade ≥ 3 adverse events in the postoperative group included lung injury (13.3%), liver dysfunction (10.0%), adrenal insufficiency (6.7%), and colitis (6.7%). No significant difference was observed in the frequency of grade ≥ 3 treatment-related adverse events between the groups, and no treatment-related deaths occurred in the postoperative group. *Conclusions:* Patients with postoperative recurrent NSCLC treated with Nivo-Ipi demonstrated significantly longer overall survival compared to those with inoperable NSCLC. Given its favorable efficacy and acceptable toxicity profile, postoperative recurrent disease may warrant consideration as a stratification factor in clinical trials for advanced NSCLC. Nivo-Ipi therapy could serve as a preferred first-line treatment option for patients with postoperative recurrent NSCLC.

## 1. Introduction

Lung cancer is one of the most common malignancies worldwide and remains the leading cause of cancer-related mortality. According to the latest estimates, lung cancer accounted for 18% of all cancer-related deaths globally in 2020, with 2.2 million new cases and 1.8 million deaths [1]. Surgical resection remains the gold standard for treating early-stage non-small cell lung cancer (NSCLC), offering the highest likelihood of a cure and significantly enhancing survival rates. Nevertheless, even after complete resection, 50–60% of patients with stages I–IIIA NSCLC eventually experience a recurrence of the disease, which often proves fatal [2,3]. Postoperative recurrence of NSCLC is rarely curable, with a reported median survival time of 8.1–17.7 months following relapse [4,5]. Furthermore, in patients who have undergone complete resection, post-progression survival has been shown to have a greater impact on overall survival (OS) than relapse-free survival (RFS) in cases where the NSCLC is negative for or has an unknown status of driver gene mutations or translocations [6]. Optimal treatment strategies for postoperative recurrence must be individualized to alleviate symptoms, preserve quality of life, and delay disease progression.

Nivolumab, a fully human monoclonal antibody targeting programmed death-1 (PD-1), and ipilimumab, a fully human monoclonal antibody targeting cytotoxic T-lymphocyte-associated protein 4 (CTLA-4), are immune checkpoint inhibitors (ICIs) with unique yet synergistic mechanisms of action. Nivolumab works by reactivating antitumor T-cell responses [7,8,9], whereas ipilimumab induces de novo antitumor T-cell responses and increases the population of memory T-cells [10,11,12]. The combination therapy of nivolumab and ipilimumab (Nivo-Ipi) has demonstrated long-lasting survival benefits in multiple advanced solid tumors, including renal cell carcinoma, esophageal squamous cell carcinoma, melanoma, and unresectable pleural mesothelioma [13,14,15,16]. Regarding NSCLC, the efficacy and safety of Nivo-Ipi combination therapy in advanced NSCLC were established in the phase 3 CheckMate 227 study [17,18]. Based on the results of this study, the regimen was approved in Japan in November 2020. As a first-line treatment, Nivo-Ipi combination therapy is extensively utilized for advanced NSCLC in patients without targetable genetic alterations in the *epidermal growth factor receptor (EGFR)* or *anaplastic lymphoma kinase (ALK)*. This therapy has demonstrated robust efficacy across various histological subtypes and PD-L1 expression levels.

Therefore, in clinical practice, Nivo-Ipi therapy is frequently administered to patients with postoperative recurrent NSCLC, following similar principles as treatment for patients with advanced or metastatic NSCLC. While some studies suggest that ICIs provide similar benefits in postoperative and inoperable NSCLC patients, others indicate that differences in tumor burden and immune microenvironment may influence treatment response. The impact of prior surgical resection on ICI efficacy remains an area of debate. However, whether the clinical benefit of Nivo-Ipi therapy differs between patients with postoperative recurrent NSCLC and those with inoperable stage III/IV NSCLC at diagnosis remains unclear. Therefore, the objective of this retrospective study was to evaluate the efficacy and safety of Nivo-Ipi therapy in these patient populations. Our findings suggest that Nivo-Ipi therapy may offer a survival benefit for patients with postoperative recurrent NSCLC, supporting its role as a viable first-line treatment option in this setting.

## 2. Materials and Methods

### 2.1. Patients

This retrospective study assessed the efficacy and safety of first-line Nivo-Ipi therapy in patients with NSCLC across four Japanese medical institutions between December 2020 and November 2022. The research protocol was approved by the Institutional Review Board of the International Medical Center at Saitama Medical University (Approval No. 2023-080). As this was a retrospective study using previously collected medical records, pre-registration was not required. The requirement for written informed consent was waived owing to the retrospective nature of the study. The inclusion criteria for this study were as follows: (1) histologically or cytologically confirmed NSCLC at inoperable stage III/IV or postoperative recurrent disease and (2) administration of Nivo-Ipi as the sole first-line treatment for recurrent disease following surgery, irrespective of prior adjuvant chemotherapy. Initially, 184 patients who received first-line Nivo-Ipi therapy were identified. Of these, 161 patients were included in the analysis after excluding 23 patients who had relapsed following (chemo)radiotherapy. The final cohort comprised 30 patients with postoperative recurrent NSCLC (postoperative group) and 131 patients with inoperable stage III/IV NSCLC at diagnosis (inoperable group) (Figure S1). However, patients with a single metastasis were not considered for reoperation because the decision for reoperation was at the discretion of each attending physician, although systemic drug therapy is considered appropriate as a treatment for recurrence. All pretreatment and treatment parameters were compared between the postoperative group and the inoperable group. The pathological diagnosis and staging of NSCLC were established in accordance with the 2015 World Health Organization classification and the 8th edition of the tumor–node–metastasis (TNM) staging system [19]. TNM staging was determined based on the findings from physical examinations, plain chest radiography, computed tomography of the thorax and abdomen, bone scintigraphy, 18F-fluorodeoxyglucose positron emission tomography (FDG-PET), and brain magnetic resonance imaging (MRI). Patient data—including baseline characteristics, responses to first-line Nivo-Ipi therapy, and AEs—were extracted by conducting a comprehensive medical chart review at each participating institution.

PD-L1 expression in formalin-fixed tumor specimens was evaluated using the commercially available immunohistochemistry kit, 22C3 pharmDx assay (Dako, Carpinteria, CA, USA) [20]. This study also included patients who had no prior testing. Biopsy samples collected either at the time of cancer diagnosis or before the initiation of Nivo-Ipi therapy were retrieved from institutional archives. However, PD-L1 expression could not be assessed in certain patients.

Patients who had previously received ICI therapy, including Nivo-Ipi, were excluded from the study population analysis. Intravenous nivolumab at doses of 240 mg or 360 mg every two or three weeks, respectively, were administered. Regarding ipilimumab, the dose administered was 1 mg/kg every six weeks intravenously, in accordance with the product labeling guidelines. However, some patients’ initial treatment consisted of Nivo-Ipi which was later revised by discontinuing intermittent ipilimumab owing to the AEs, according to the physician’s clinical judgment. Thereafter, the first-line Nivo-Ipi therapy was continued until disease progression, intolerable toxicity, or patient consent withdrawal. In cases of disease progression occurring during first-line treatment, patients were allowed to explore alternative therapeutic options.

### 2.2. Treatment Efficacy and Evaluation of AEs

The best overall response and maximum tumor shrinkage were recorded as tumor responses. Radiographic tumor responses were assessed based on the Response Evaluation Criteria in Solid Tumors, version 1.1 (RECIST v1.1) [21]. Tumor response to treatment was categorized into five groups: complete response (CR), partial response (PR), stable disease (SD), progressive disease (PD), and not evaluable (NE). Assessments of response were conducted by the attending physicians, with no centralized review process implemented.

AEs associated with Nivo-Ipi therapy were evaluated in accordance with version 5.0 of the Common Terminology Criteria for Adverse Events (CTCAEs).

### 2.3. Statistical Analyses

Categorical variables were analyzed using Fisher’s exact test, while continuous variables were assessed using Welch’s *t*-test. Progression-free survival (PFS) was defined as the time from Nivo-Ipi initiation to PD or death. OS was calculated from the first day of Nivo-Ipi treatment until death or censoring at the last follow-up. RFS was defined as the time from surgery to the first documented relapse or death from any cause. Potential clinical factors associated with PFS and OS were evaluated using univariate and multivariate analyses. The Cox proportional hazards model was applied with a stepwise regression procedure to identify prognostic factors. Hazard ratios (HRs) and 95% confidence intervals (CIs) were calculated. Survival curves were generated using the Kaplan–Meier method and compared between the 2 groups using the log-rank test. A two-tailed *p*-value of <0.05 was considered statistically significant. All statistical analyses were conducted using JMP software (version 11.0; SAS Institute, Cary, NC, USA).

## 3. Results

### 3.1. Patient Characteristics

The current analysis included a total of 161 patients, comprising 30 (18.6%) with postoperative recurrent NSCLC and 131 (81.4%) with inoperable stage III/IV NSCLC. The baseline characteristics, stratified by group, are presented in Table 1. The median interval from surgical resection of the primary disease to the initiation of Nivo-Ipi therapy was 15.4 months (range: 4.7–82.1 months). The distributions of age, sex, smoking history, histological subtype, PD-L1 TPS, and metastatic sites were similar between the two groups. However, patients in the postoperative group demonstrated a significantly better ECOG-PS compared to those in the inoperable group (*p* = 0.003).

The clinical factors in patients with postoperative recurrence are summarized in Table S1. Among the postoperative group, pathological stage I, II, and III disease was observed in 13 (43.3%), 5 (16.7%), and 12 (40.0%) patients, respectively. Surgical procedures included partial or wedge resection in 6 patients (20.0%), lobectomy in 23 patients (76.7%), and total pneumonectomy in 1 patient (3.3%). Adjuvant chemotherapy was administered to 12 patients (40.0%) in the postoperative group. Following surgical resection, the number of metastatic sites was one in 8 patients (26.7%), two in 16 patients (53.3%), and 3 or more in 6 patients (20.0%). The median RFS was 13.9 months (95% CI: 10.6–19.5 months).

As of the data cutoff date (31 October 2023), 6 patients in the postoperative group and 20 patients in the inoperable group were continuing Nivo-Ipi therapy.

### 3.2. Treatment Response and Survival

**Figure 1:**
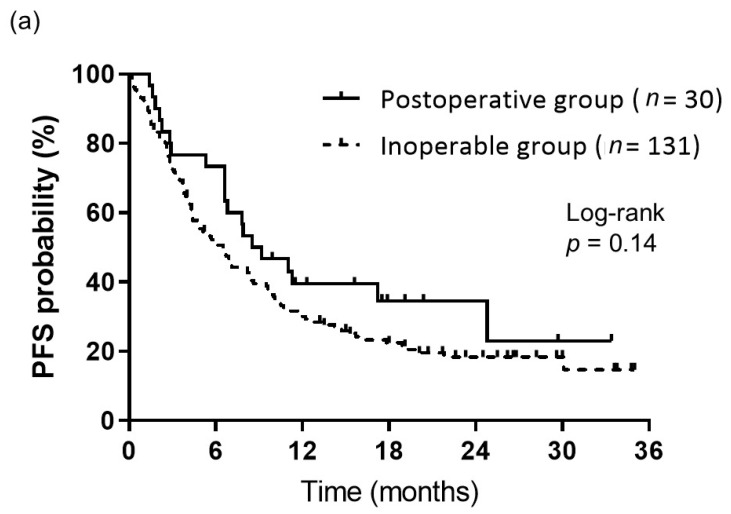

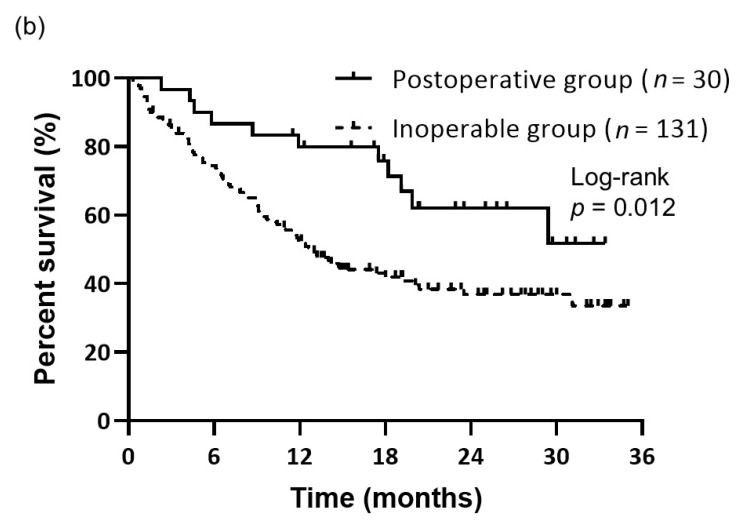
Kaplan–Meier curves of survival. (**a**) PFS of patients in the postoperative (*n* = 30) and inoperable (*n* = 131) groups. PFS did not significantly differ between the groups (median: 8.8 months for the postoperative group vs. 6.5 months for the inoperable group, *p* = 0.14). (**b**) OS of patients in the postoperative (*n* = 30) and inoperable (*n* = 131) groups. OS differed significantly between the groups (median: not reached for the postoperative group vs. 13.0 months for the inoperable group, *p* = 0.012).

The objective responses to first-line Nivo-Ipi therapy are summarized in Table 2. During the follow-up period, the postoperative group demonstrated response outcomes as follows: 1 patient achieved a CR, 10 achieved a PR, 11 had SD, 7 exhibited PD, and 1 was NE. In comparison, the inoperable group recorded 0 cases of CR, 42 of PR, 44 of SD, 38 of PD, and 7 of NE. The ORR for the postoperative group was comparable to that of the inoperable group (36.7% vs. 32.1%, *p* = 0.67). The median follow-up duration from the initiation of Nivo-Ipi therapy was 13.1 months. There was no significant difference in median PFS between the postoperative and inoperable groups (8.8 months vs. 6.5 months, *p* = 0.14) (Figure 1a). However, the median OS was notably longer in the postoperative group compared to the inoperable group (not reached vs. 13.0 months, *p* = 0.012) (Figure 1b).

At the time of data cutoff, 11 of the 30 patients (36.7%) in the postoperative group and 78 of the 131 patients (59.5%) in the inoperable group had died during the follow-up period.

### 3.3. Prognostic Factors

The prognostic significance of various PFS and OS metrics was evaluated within the postoperative group (Table 3). Univariate analysis demonstrated that body mass index (BMI) was significantly associated with PFS. However, no statistically significant factors were found for OS. Nivo-Ipi therapy showed an independent association with enhanced PFS in multivariate analysis, even after adjusting for various clinical factors, including BMI (*p* = 0.01). Notably, BMI significantly modified the effect of Nivo-Ipi therapy on median PFS, with patients having a BMI of <22 showing a longer PFS compared to those with a BMI of ≥22 (not reached vs. 6.6 months, log-rank *p* = 0.0091). However, in the multivariate analysis of OS, none of the clinical factors were identified as independent prognostic factors.

### 3.4. Safety

The primary AEs observed during treatment are outlined in Table 4, with skin rash identified as the most frequently occurring treatment-related AE. In the postoperative group, 45.2% of patients experienced a skin rash of any grade, while 3.3% developed a grade 3–4 skin rash. In the inoperable group, 35.9% of patients experienced a skin rash of any grade, and 3.1% developed a grade 3–4 skin rash. Liver dysfunction was reported in 36.7% of patients in the postoperative group and 28.2% in the inoperable group. Among treatment-related AEs of grade 3 or higher, lung injury was the most frequently observed, occurring in 13.3% of the postoperative group and 10.7% of the inoperable group. Other grade 3–4 AEs included adrenal insufficiency and colitis, each occurring in 6.7% of patients in the postoperative group. Patients who developed these AEs received standard supportive care. There was no statistically significant difference in the overall incidence of treatment-related AEs between the two groups for any grade. Treatment discontinuation owing to AEs occurred in 7 patients (23.3%) in the postoperative group and 33 patients (25.2%) in the inoperable group. One patient in the inoperable group experienced a treatment-related fatal lung injury, whereas no treatment-related deaths occurred in the postoperative group.

## 4. Discussion

In our study, the ORR of Nivo-Ipi treatment was comparable between the two groups. Although there was a trend toward longer PFS in the postoperative group, the difference was not statistically significant. However, OS was significantly longer in the postoperative group than in the inoperable group.

According to the National Comprehensive Cancer Network (NCCN) guidelines, recurrence after definitive therapy—including postoperative recurrence—is considered systemic disease progression, and treatment options include systemic chemotherapy as part of the management for recurrence and metastasis [22]. Specifically, for patients with advanced NSCLC with a good PS, drug therapies (cytotoxic anticancer agents, molecularly targeted therapies, and ICIs) are considered appropriate. When formulating the treatment strategy, it is important to first screen for driver gene mutations/translocations in the tumor, and if positive, targeted therapy specific to each driver gene should be considered, at the appropriate treatment stage. However, when driver gene mutations/translocations are negative, immunotherapy should be considered, except when ICI administration is contraindicated. The ECOG report reinforces the notion that patients with postoperative recurrence tend to have a more favorable prognosis compared to those with stage IV disease [23]. However, this report does not include patients treated with ICIs. Several studies have reported improved survival outcomes in postoperative patients compared to inoperable patients receiving ICIs [24,25]. Notably, there are currently no published studies focusing solely on Nivo-Ipi treatment, making our study unique in this regard. Yuasa et al., in a study of 87 patients, including 35 and 52 who received ICIs as first- and second-line treatment or later, respectively, reported that patients receiving ICI as first-line treatment had a better prognosis than those receiving ICI as second-line or later therapy. The PFS and OS were 6.3 months and 25.0 months, respectively, for first-line treatment, compared to 2.8 months and 14.5 months, respectively, for later-line treatment [24]. Motono et al. examined patients with postoperative recurrence of NSCLC and reported the following findings [25]: the 3-year OS rate after recurrence was 79.3% in the EGFR-TKI group (*n* = 23), 69.5% in the ICI monotherapy group (*n* = 31), and 43.7% in the cytotoxic chemotherapy group (*n* = 10). Although there was no significant difference in OS between the EGFR-TKI and ICI groups (*p* = 0.14) or between the ICI and cytotoxic chemotherapy groups (*p* = 0.23), OS was significantly longer in the EGFR-TKI group compared to the cytotoxic chemotherapy group (*p* < 0.01). Based on these findings, the study concluded that EGFR-TKIs and ICIs are effective treatment options for recurrent NSCLC following surgery.

Although findings remain unclear, several hypotheses may explain the observed outcomes. Tumor heterogeneity and tumor burden could play a role, as tumor heterogeneity contributes to resistance, and small subpopulations of tumor cells may acquire or inherently possess adaptive features that enable them to survive under selective drug pressure [26,27]. Most patients with postoperative recurrent NSCLC undergo regular follow-up after surgical resection, potentially resulting in a lower tumor burden compared to patients with inoperable stage III/IV NSCLC at diagnosis. These differences in tumor heterogeneity and burden may be associated with the improved PFS and OS observed in the postoperative group [26,28].

Regarding the results of univariate and multivariate analyses in the postoperative group, BMI was identified as an independent prognostic factor for PFS, whereas no significant prognostic factors were found for OS. This may be attributed to the small sample size of 30 patients, which could have limited the statistical power to detect significant associations. Additionally, we set the BMI threshold at 22 kg/m^2^, which corresponds to the ideal BMI for Japanese individuals [29]. The suitability of this cutoff value remains debatable because it is influenced by ethnic and population-specific variations. Moreover, BMI is shaped by a range of factors, such as genetic predisposition, inherent body structure, tumor progression, cachexia, and psychological conditions. These confounding variables complicate efforts to determine a definitive, independent correlation between BMI and treatment outcomes or survival, even with the application of multivariable analysis.

With respect to the AEs, their incidence was similar between the two cohorts in this study. The incidence of AEs resulting in treatment discontinuation was consistent with previously documented data [17]. While a direct comparison between the prospective CheckMate 227 trial and this retrospective study is not feasible, our findings indicate that the toxicity profile of Nivo-Ipi therapy in patients with postoperative recurrence closely mirrors that observed in the CheckMate 227 trial [17]. While no grade 5 AEs were observed among patients with postoperative recurrence, the sample size was insufficient to establish a definitive correlation between Nivo-Ipi therapy and treatment-related mortality. Additionally, the AE profile of Nivo-Ipi therapy in these patients aligned with previously reported data. However, the incidence of lung injury across all grades was notably high at 26.7%, with grade 3 or higher pneumonitis observed in 13.3% of cases. All affected patients recovered following supportive care, including corticosteroid treatment. Although no treatment-related fatalities occurred, the frequency of pneumonitis reported in this study exceeded that documented in a prospective study, which indicated rates of 8.3% for any grade and 3.3% for grade 3 or higher [17]. These discrepancies emphasize the inherent limitations of clinical trials in comprehensively capturing drug safety profiles and highlight the critical role of real-world data in assessing treatment-related toxicities [30]. Based on our findings, treatment-related AEs associated with Nivo-Ipi therapy in patients with postoperative recurrence were manageable, with no specific AEs observed that were distinct from those observed in inoperable patients.

There were some limitations to this study. First, the sample sizes of the postoperative and inoperable groups were imbalanced. The analysis included only 30 patients in the postoperative cohort, which significantly limits the statistical power for subgroup analyses and might have affected the reliability in the observed differences. Larger studies are necessary to validate our findings and assess their generalizability to real-world clinical settings. Second, as a retrospective observational study, this analysis inherently carries the limitations of retrospective data collection. Disease progression and AE assessments may have differed from those in prospective trials. Furthermore, the retrospective nature of the study inherently limits the ability to infer causality and introduces potential biases, including selection and information biases. Future prospective studies are necessary to validate these findings. Third, there is a lack of stratified analysis by key prognostic factors. The absence of stratified analyses based on well-established prognostic variables (including PD-L1 expression, oncogenic mutation status, and ECOG-PS) may limit an accurate interpretation of treatment outcomes. Including stratified analyses could provide more nuanced insights into which subpopulations would benefit the most. Fourth, the fact that the study involved ethnic and regional homogeneity is a limitation. This is because the study population was exclusively from Japan; thus, the generalizability and applicability of the findings to populations with different ethnic backgrounds, environmental exposures, and healthcare systems may be limited. Study cohorts that are more diverse are necessary to enhance broader generalizability. Fifth, Nivo-Ipi therapy could be delayed or omitted at the discretion of the treating physician, introducing potential treatment variability. To address potential sources of bias, we sampled all consecutive patients treated at our institutions and ensured comprehensive documentation through meticulous medical chart reviews. Additionally, the administration of first-line Nivo-Ipi therapy and the choice of subsequent treatments were left to the discretion of the attending physicians. This may have introduced selection bias, potentially affecting survival outcomes following subsequent therapies. Regarding survival outcomes, OS in our study was relatively shorter compared to that observed in the Nivo-Ipi cohort of the CheckMate 227 trial. This discrepancy may be attributable to an immature observation period in our analysis. Future studies with extended follow-up durations will be necessary to assess long-term survival outcomes with greater accuracy. The fact that the median OS was not reached in the postoperative group suggests the need for longer follow-up. Extended observation periods are crucial to accurately assess survival benefits and late AEs.

Overall, our results indicate that OS was significantly longer in patients with postoperative recurrent NSCLC treated with Nivo-Ipi than that in the inoperable group. Nivo-Ipi therapy demonstrated favorable effectiveness and an acceptable toxicity profile in the postoperative group. The results indicate that postoperative recurrent disease could serve as a stratification criterion in clinical trials targeting advanced NSCLC. Additionally, Nivo-Ipi therapy could be a preferred standard treatment modality for patients with postoperative recurrent NSCLC.

## 5. Conclusions

Patients with postoperative recurrent NSCLC treated with Nivo-Ipi demonstrated significantly longer overall survival compared to those with inoperable NSCLC. Given its favorable efficacy and acceptable toxicity profile, postoperative recurrent disease may warrant consideration as a stratification factor in clinical trials for advanced NSCLC. Nivo-Ipi therapy could serve as a preferred first-line treatment option for patients with postoperative recurrent NSCLC.

## Figures and Tables

**Table 1:** Patient characteristics.

Characteristics	Total (*n* = 161) (%)	Postoperative Group (*n* = 30) (%)	Inoperable Group (*n* = 131) (%)	*p*-Value
Sex				
Male	128 (79.5)	25 (83.3)	103 (78.7)	0.63
Female	33 (20.5)	5 (16.7)	28 (21.3)	
Median age at treatment (years) [range]	72 (46–85)	74 (46–82)	72 (46–85)	0.20 *
Performance status (PS)				
0	51 (33.2)	20 (66.7)	31 (23.7)	**0.003**
1	81 (50.5)	10 (33.3)	71 (54.2)	
≥2	29 (16.3)	0	29 (22.1)	
Smoking history				
Yes	143 (88.8)	26 (86.7)	117 (89.3)	0.75
No	18 (11.2)	4 (13.3)	14 (10.7)	
Clinical stage at diagnosis				
I	13 (8.1)	13 (43.3)	-	-
II	5 (3.1)	5 (16.7)	-	
III	23 (14.3)	12 (40.0)	11 (8.4)	
IV	120 (74.5)	-	120 (91.6)	
Histology				
Adenocarcinoma	95 (59.0)	20 (66.7)	75 (57.3)	0.11
Squamous cell carcinoma	45 (28.0)	10 (33.3)	35 (26.7)	
Carcinoma	6 (3.7)	0	6 (4.6)	
Not otherwise specified	16 (9.9)	0	16 (12.2)	
PD-L1 TPS				
<1	76 (47.2)	12 (40)	64 (48.9)	0.57
1–49	61 (37.9)	15 (50)	46 (35.1)	
≥50	15 (9.3)	2 (6.7)	13 (9.9)	
Unknown	9 (5.6)	1 (3.3)	8 (6.1)	
Metastatic site				
Brain	24 (14.9)	7 (23.3)	17 (13.0)	0.16
Liver	19 (11.8)	2 (6.7)	17 (13.0)	0.53
Bone	53 (32.9)	5 (16.7)	48 (36.6)	0.05
Body fluids (pleural effusion or ascites)	45 (28.0)	4 (13.3)	41 (31.3)	0.07
BMI (kg/m^2^)				
Median [range]	22.0 (13.4–42.0)	23.4 (18.6–42.0)	21.6 (13.4–30.5)	0.001 *
Number of cycles nivolumab plus ipilimumab administered				
Median (range)	2 (1–20)	5 (1–13)	2 (1–20)	0.0088 *
Continuing administration of nivolumab plus ipilimumab at data cutoff				
Yes	26	6	20	0.58
No	135	24	111	
Survival status at data cut-off				
Death	89	11	78	**0.0264**
Alive	72	19	53	

Fisher’s exact test. * Welch’s *t*-test. Bold font indicates statistically significant differences. PD-L1, programmed cell death ligand 1; TPS, tumor proportion score; BMI, body mass index.

**Table 2:** Treatment response.

	Total (*n* = 161)	Postoperative Group (*n* = 30)	Inoperable Group (*n* = 131)	*p*-Value #
Response				
Complete response	1	1	0	
Partial response	52	10	42	
Stable disease	55	11	44	
Progressive disease	45	7	38	
Not evaluated	8	1	7	
Response rate (%) (95% CI)	32.9 (26.1–40.5)	36.7 (21.8–54.5)	32.1 (24.6–40.4)	0.67
Disease control rate (%) (95% CI)	67.1 (59.5–73.9)	73.3 (55.3–86.0)	65.7 (57.2–73.2)	0.52

# Comparison between the postoperative and inoperable groups. CI, confidence interval.

**Table 3:** Univariate analyses of progression-free survival (PFS) and overall survival (OS) in the postoperative group.

Variables	Median PFS	Univariate Analysis			Multivariate Analysis			Median OS	Univariate Analysis			Multivariate Analysis		
	(Months)	HR	95% CI	*p*-Value	HR	95% CI	*p*-Value	(Months)	HR	95% CI	*p*-Value	HR	95% CI	*p*-Value
Sex														
Male (*n* = 25)/female (*n* = 5)	7.9/17.2	1.54	0.52–4.49	0.48				29.4/26.2	2.12	0.44–10.05	0.49			
Age at the start of Nivo-Ipi (years)														
<75 (*n* = 17)/≥75 (*n* = 13)	7.9/17.2	1.2	0.49–2.91	0.67				NR/NR	0.59	0.16–2.07	0.35			
Histology														
Adenocarcinoma (*n* = 20)/non-adenocarcinoma (*n* = 10)	8.5/9.9	1.1	0.43–2.82	0.83				NR/NR	0.75	0.20–2.74	0.65			
Smoking history														
Yes (*n* = 26)/no (*n* = 4)	8.2/17.2	1.83	0.56–5.89	0.70				29.4/NR	-	−1.0–1.0	0.19			
PD-L1 TPS (%)														
<1% or unknown (*n* = 13)/≥1% (*n* = 17)	NR/7.9	0.57	0.23–1.39	0.23	0.45	0.15–1.20	0.11	29.4/NR	1.62	0.46–5.66	0.41	2.06	0.56–7.31	0.26
Postoperative adjuvant chemotherapy														
Yes (*n* = 12)/no (*n* = 18)	19.4/8.1	0.57	0.23–1.38	0.23				NR/29.4	0.63	0.18–2.14	0.49			
Intracranial metastases at the start of Nivo-Ipi														
Yes (*n* = 6)/no (*n* = 24)	8.5/9.9	1.48	0.47–4.60	0.42				NR/29.4	0.71	0.17–2.88	0.66			
Liver metastases at the start of Nivo-Ipi														
Yes (*n* = 2)/no (*n* = 28)	19.2/NR	1.25	0.13–12.12	0.82				19.2/NR	1.28	0.13–12.58	0.81			
Bone metastases at the start of Nivo-Ipi														
Yes (*n* = 5)/no (*n* = 25)	6.8/9.2	1.39	0.41–4.71	0.54				NR/NR	1.51	0.25–8.90	0.58			
Body fluids (pleural effusion or ascites) at the start of Nivo-Ipi														
Yes (*n* = 3)/no (*n* = 27)	5.3/9.2	1.12	0.24–5.19	0.87				19.1/NR	2.02	0.27–14.60	0.35			
Relapse-free survival														
<12 months (*n* = 10)/≥12 months (*n* = 20)	2.85/11.0	1.93	0.66–5.60	0.14	1.94	0.68–5.25	0.20	17.5/NR	2.67	0.66–10.73	0.08	2.3	0.63–8.12	0.19
BMI, kg/m^2^														
<22 (*n* = 9)/≥22 (*n* = 21)	NR/6.6	0.23	0.09–0.56	0.0091	0.24	0.05–0.75	0.01	NR/29.4	0.17	0.05–0.59	0.05	0.18	0.009–1.01	0.05

PFS, progression-free survival; OS, overall survival; HR, hazard ratio; CI, confidence interval; Nivo-Ipi, nivolumab plus ipilimumab; PD-L1, programmed cell death ligand 1; TPS, tumor proportion score; BMI, body mass index.

**Table 4:** Treatment-related adverse events.

	All Patients (*n* = 161)				Postoperative Group (*n* = 30)				Inoperable Group (*n* = 131)				
Adverse Event	Any Grade	%	Grade ≥ 3	%	Any Grade	%	Grade ≥ 3	%	Any Grade	%	Grade ≥ 3	%	*p*-Value #
Resulted in discontinuation	40	24.8	26	16.1	7	23.3	4	13.3	33	25.2	22	16.8	>0.99
Resulted in death	-	-	1	0.6	-	-	0	0	-	-	1	0.8	>0.99
Treatment-related													
Skin rash	61	37.9	5	3.1	14	45.2	1	3.3	47	35.9	4	3.1	0.30
Liver dysfunction	48	29.8	15	9.3	11	36.7	3	10.0	37	28.2	12	9.2	0.38
Thyroid dysfunction	35	21.7	0	0	10	33.3	0	0	25	19.1	0	0	0.14
Lung injury	32	19.9	18	11.2	8	26.7	4	13.3	24	18.3	14	10.7	0.35
Adrenal insufficiency	31	19.3	16	9.9	6	20.0	2	6.7	25	19.1	14	10.7	>0.99
Elevated creatinine levels	27	16.8	0	0	6	20.0	0	0	21	16.0	0	0	0.59
Colitis	27	16.8	11	6.8	6	20.0	2	6.7	21	16.0	9	6.9	0.59
Fever	14	8.7	1	0.6	2	6.7	0	0	12	9.2	1	0.8	>0.99
Myositis	12	7.5	3	1.9	3	10.0	1	3.3	9	6.9	2	1.5	0.70
Arthritis	8	4.3	0	0	0	0	0	0	8	6.1	0	0	0.35
Type 1 diabetes	2	1.3	1	0.6	1	3.3	0	0	1	0.8	1	0.8	0.34

# Comparison of treatment-related adverse events of any grade between the postoperative and inoperable groups.

## Data Availability

The data supporting this study are available upon reasonable request from the corresponding author. However, access is restricted due to ethical and privacy constraints.

## Supplementary Materials

The following supporting information can be downloaded at: https://www.mdpi.com/article/10.3390/medicina61060994/s1, Figure S1: Diagram showing patient selection. The patients treated with nivolumab + ipilimumab treatment between December 2020 and November 2022; Table S1: Clinical factors in patients with postoperative recurrence.

## Abbreviations

The following abbreviations are used in this manuscript:

AE	Adverse event
AL	Anaplastic lymphoma
BMI	Body mass index
CI	Confidence interval
CTCAE	Common Terminology Criteria for Adverse Events
CTLA-4	Cytotoxic T-lymphocyte-associated protein 4
EGFR	Epidermal growth factor receptor
ECOG-PS	Eastern Cooperative Oncology Group performance status
ICI	Immune checkpoint inhibitor
HR	Hazard ratio
NCCN	National Comprehensive Cancer Network
NE	Not evaluable
NSCLC	Non-small cell lung cancer
Nivo-Ipi	Nivolumab plus ipilimumab
OS	Overall survival
ORR	Objective response rate
PD	Progressive disease
PD-1	Programmed death-1
PD-L1	Programmed death-ligand 1
PFS	Progression-free survival
PR	Partial response
RFS	Relapse-free survival
RECIST	Response Evaluation Criteria in Solid Tumors
SD	Stable disease
TNM	Tumor–node–metastasis
