# Supplementary material for: Efficacy and Safety of First-Line Nivolumab Plus Ipilimumab in Patients with Postoperative Recurrent and Inoperable Non-Small Cell Lung Cancer: A Real-World Retrospective Observational Study
Source: Medicina (Kaunas). 2025 May 27;61(6):994. doi: 10.3390/medicina61060994 (PMC12195084; doi:10.3390/medicina61060994)
Supplement: Supplementary file 1 [file medicina-61-00994-s001.zip › medicina-3570947-supplementary.pdf]

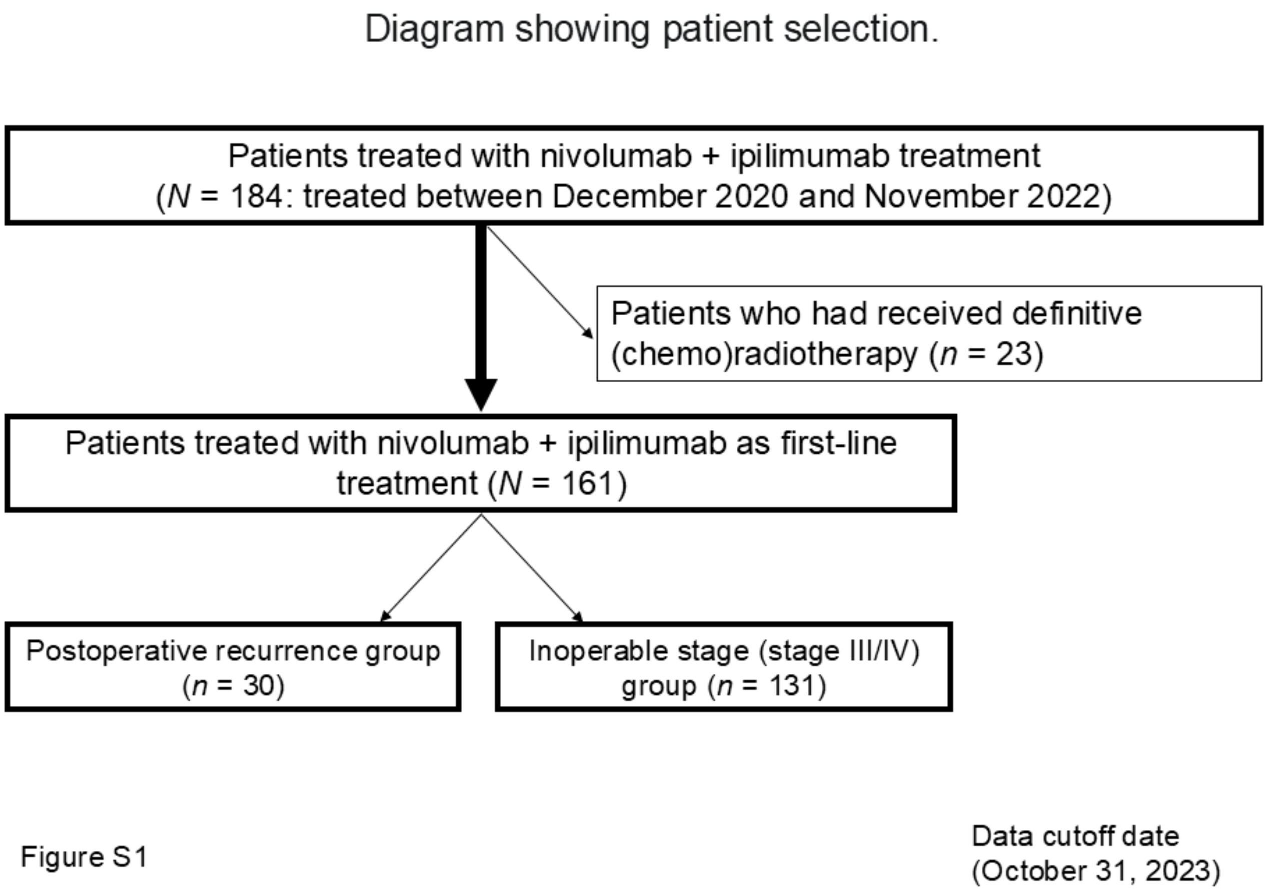

Figure S1. Diagram showing patient selection. The patients treated with nivolumab + ipilimumab treatment between December 2020 and November 2022.

Supplement Table 1. Clinical factors in patients with postoperative recurrence

Characteristics		Postoperative group (*n*=30)	(%)
T factor			
	1	11	36.7
	2	13	43.3
	3	5	16.7
	4	1	3.3
N factor			
	0	16	53.3
	1	4	13.3
	2	10	33.3
Clinical stage at diagnosis			
	IA	7	23.0
	IB	6	20.0
	IIA	1	3.3
	IIB	4	13.0
	IIIA	9	30.0
	IIIB	3	10.0
Surgical procedure			
Partial or wedge resection		6	20.0
Lobectomy		23	76.7
Total pneumonectomy		1	3.3
Postoperative adjuvant chemotherapy			

Yes	12	40.0
No	18	60.0
Metastatic site		
Brain	7	23.3
Liver	2	6.7
Bone	5	16.7
Body fluids (pleural effusion or ascites)	4	13.3
Number of metastatic sites		
1	8	26.7
2	16	53.3
3	4	13.3
4	2	6.7
The interval from surgical resection for the primary disease to the start of nivolumab+ipilimumab		
Median (months) [range]	15.4 (4.7-82.1)	
